# Supplementary material for: Synthesis, Biological Activity, ADME and Molecular Docking Studies of Novel Ursolic Acid Derivatives as Potent Anticancer Agents
Source: Int J Mol Sci. 2023 May 17;24(10):8875. doi: 10.3390/ijms24108875 (PMC10219251; doi:10.3390/ijms24108875)
Supplement: Supplementary file 1 [file ijms-24-08875-s001.zip › ijms-2346037-supplementary.pdf]

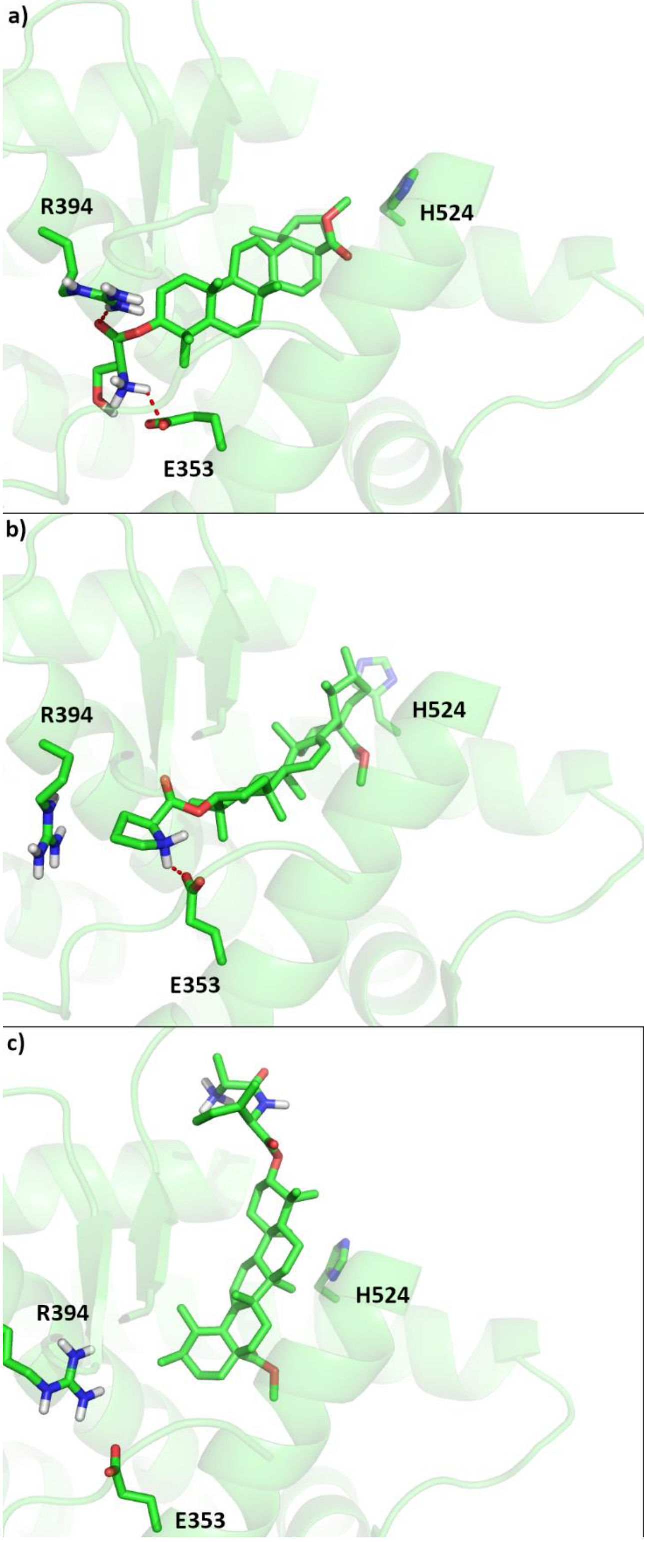

Figure S1. Predicted conformations and orientations of selected ligands in the estrogen receptor binding pocket; a) compound **4c**; b) compound **7a**; c) compound **10a**.
